# Ablation of atypical atrial flutters using ultra high density-activation sequence mapping

**DOI:** 10.1007/s10840-016-0207-5

**Published:** 2016-11-10

**Authors:** Roger A. Winkle, Ryan Moskovitz, R. Hardwin Mead, Gregory Engel, Melissa H. Kong, William Fleming, Rob A. Patrawala

**Affiliations:** 1 Silicon Valley Cardiology, 1950 University Avenue, Suite 160, E. Palo Alto, CA 94303 USA; 2 Sequoia Hospital, Redwood City, CA USA; 3 St. Jude Medical, Inc., St. Paul, MD USA

**Keywords:** Atrial flutter, Atrial flutter ablation, Activation mapping, Atypical atrial flutter, Left atrial flutter

## Abstract

**Purpose:**

The purpose of this study was to evaluate ultra high density-activation sequence mapping (UHD-ASM) for ablating atypical atrial flutters.

**Methods:**

For 23 patients with 31 atypical atrial flutters (AAF), we created UHD-ASM.

**Results:**

Demographics age = 65.3 ± 8.5 years, male = 78%, left atrial size = 4.66 ± 0.64 cm, redo ablation 20/23(87%). AAF were left atrial in 30 (97%). For each AAF, 1273 ± 697 points were used for UHD-ASM. Time to create and interpret the UHD-ASM was 20 ± 11 min. For every AAF, the entire circuit was identified. Thirty (97%) were macroreentry. AAF cycle length was 267 ± 49 ms, and the circuit length was 138 ± 38 mm (range 35–187). Macroreentry atrial flutters took varied pathways, but each had an area of slow conduction (ASC) averaging 16 ± 6 mm (range 6–29) in length. Entrainment was not utilized. We targeted the ASC and ablation terminated AAF directly in 19/31 (61.3%) and altered AAF activation in 7/31 (22.6%), all of which terminated directly with additional mapping/ablation. AAF degenerated to atrial fibrillation in 2/31 (6.5%) with RF and could not be reinduced after ASC ablation. Median time from initial ablation to AAF termination was 64 s. Thus, 28/31 (90.3%) terminated with RF energy and/or could not be reinduced after ASC ablation. At 1 year of follow-up, 77% were free of atrial tachycardia or atrial flutter and 61% were free of all atrial arrhythmias.

**Conclusions:**

Using rapidly acquired UHD-ASM, the entire AAF circuit as well as the target ASC could be identified. Most AAF were left atrial macroreentry. Ablation of the ASC or microreentry focuses directly terminated or eliminated AAF in 90.3% without the need for entrainment mapping.

**Electronic supplementary material:**

The online version of this article (doi:10.1007/s10840-016-0207-5) contains supplementary material, which is available to authorized users.

## Introduction

For decades, there was a scientific debate as to whether or not typical atrial flutter was focal or reentrant [1, 2]. With the introduction of newer computer-driven mapping systems, it became clear that the vast majority of typical atrial flutters were reentrant and involved the right atrial caval-tricuspid isthmus in either a counterclockwise or clockwise rotational pattern. Atypical atrial flutters, however, have proven more challenging to map and ablate [3–9]. Many of these atrial flutters occur in patients who have undergone previous medical procedures such as valvular or congenital cardiac surgery involving atriotomy, surgical Maze operations, or pulmonary vein isolation procedures. Mapping and ablation of these complex arrhythmias continue to be a challenge for clinical electrophysiologists. A combination of activation sequence mapping and entrainment [10] has commonly been utilized to understand and ablate these arrhythmias. In this study, we report our experience with the use of ultra high density-activation sequence mapping (UHD-ASM) to determine the precise arrhythmia circuit and to identify potential targets for successful ablation.

## Methods

### Patient population

The subjects were symptomatic patients undergoing ablations for atypical, non-right atrial isthmus-dependent flutter at Sequoia Hospital, Redwood City, CA, from October 4, 2013 to October 21, 2014. All signed the written informed consent. Data analysis was retrospective and approved by the Western Institutional Review Board.

### Ablation protocol

Our ablation protocol has been described previously [11]. Antiarrhythmic drugs were stopped at least five half-lives and amiodarone at least 3 months before ablation. General anesthesia was used in all, and venous access was from the right groin only. A 9F Boston Scientific (Natick, MA) Ultra Ice™ catheter guided the transseptal puncture, done using a 71-cm Baylis (Montreal, QC) NRG™ needle. The mapping and ablation catheters were both placed in the left atrium across a single transseptal puncture. All ablations were done using a Safire™ BLU™ (St Jude Medical, St. Paul, MN) open irrigated tip ablation catheter at 50 W. Patients had a femoral or radial arterial line. Our peri-procedural anticoagulation protocols have also been previously described [12].

### Mapping protocol

3D geometry and mapping were done using the St. Jude EnSite Velocity system with Precision Mapping Module. A 7F 20 electrode catheter (St. Jude Livewire™, 2-10-2 mm electrode spacing catheter) was placed around the tricuspid valve annulus with the distal poles in the coronary sinus. Following access to the left atrium, a 3D geometry was created. Patients who were in their clinical atrial arrhythmia at the start of the case had activation mapping simultaneous with geometry creation. A stable atrial bipolar electrogram, preferably from an electrode pair inside the coronary sinus, was chosen as an activation mapping reference and a reference detection algorithm was chosen to ensure consistent detection and timing of the reference electrogram. A surface ECG with a visible atrial flutter wave was added to the mapping window to confirm stability of the reference electrogram relative to the atrial flutter wave. The left edge of the mapping window was set at 60 ms ahead of the onset of the most prominent atrial flutter wave on the surface ECG, and the right edge of the mapping window was set so that at least 95% of the tachycardia cycle length was encompassed within the mapping window. Timing within the arrhythmia cycle length was divided into eight equal parts, each tagged with a different color to create an isochronal color map. These color maps are similar to traditional isochronal maps where lines are drawn to reflect zones of similar activation times. The only difference was that there are no lines drawn between the various colors. Slower conduction is indicated by a narrowing of the width of the individual color zones or a compression of the colors. A circular mapping catheter (St. Jude Reflexion Spiral™) with 20 electrodes, and a 15–25 mm variable loop was used as a roving mapping catheter. The bipolar electrograms were recorded from 1 mm electrodes (except for the tip which is 2 mm) separated by 1 mm. Up to 10 simultaneously collected bipolar electrograms were obtained at each anatomical position of the roving catheter. The detection setting was configured to automatically detect the largest amplitude bipolar peak, and the sensitivity was set to the maximum of 10 mV. This combination allowed the computer to detect and annotate the largest amplitude bipolar peak in the mapping window regardless of the voltage. In the case of double potentials or fractioned electrograms within an area of interest, the user could manually override the automatic detection to annotate the timing of the said point to the peak of the sharpest near-field component of the signal. Using this method of collecting a high density of points with a multipolar mapping catheter, the need for this manual annotation is generally minimal. However, when delineating activation in areas of dense scar and mapping locations was prone to high amplitude far-field signals, such as the anterior left superior pulmonary vein which often also has right atrial signals or the AV groove which often also has ventricular signals, manual editing may still be necessary.

The low voltage identification level was set to 0.1 mV initially to allow low voltage zones to be collected and displayed as gray colored low voltage zones on the map. This helped to prevent inaccurate timing measurements when sampling from regions of scarred or non-conductive tissue.

Following the appropriate setting of all map values, the first set of activation mapping points were collected from the coronary sinus reference catheter to store the map settings as well as the reference catheter activation sequence for the atrial arrhythmia. This allowed a set of waveform shadows to be visualized behind the live coronary sinus catheter electrograms. If a change occurred in the intracardiac activation sequence, these shadows helped the user identify that a rhythm change had taken place or that the reference catheter had moved. When either no anatomical surface model had been created or the mapping catheter was in a new area of the chamber that had not previously had geometry created, anatomical static shell points and activation mapping points were collected simultaneously. While the roving circular catheter was being moved throughout the chamber, the mapping system rapidly acquired 10 activation points at a time. During rapid acquisition, the operator monitored the signals to eliminate collection of points with noise or ventricular signal. When the full chamber was mapped, field scaling was applied, the geometry was edited and completed, and the activation map was projected onto the geometry surface to evaluate the arrhythmia circuit. Mapping points not in close proximity to the static model geometry surface were excluded from the analysis. This eliminated any far-field electrograms collected inside the chamber from being used to create the activation map of the atrial arrhythmias. Both color isochronal and propagation maps were generated. If any areas of interest in the chamber were determined to be under-sampled, additional points were added to the activation map as deemed necessary. This was most often needed in the areas of slowest conduction. In a highly diseased atrial chamber, or to determine the activation sequence through the areas of slow conduction, the low voltage level was adjusted to as low as 0.03 mV for visualization of low-amplitude signals within the areas of scar. The circuit of each macroreentrant atrial flutter was measured by tracing the leading edge of each color isochronal zone. Although we used eight color isochrones during the clinical studies, during post hoc evaluation of the circuit, we sometimes used 15 and 22 isochrones to more accurately define the atrial flutter circuit. This allowed measurement of the total length of the atrial flutter circuit. The area of slowest conduction was determined for each atrial flutter, and the length of the slow zone and time of conduction through this zone were determined. Microreentrant atrial flutter or focal atrial tachycardia was defined as an arrhythmia where the origin of the arrhythmia occurred in a very small area (<1 cm) and radiated outward from that site. At the end of each ablation, all pulmonary veins were checked to make certain that they were isolated with both entrance and exit block and any which were still connected were re-ablated to complete PV isolation. Arrhythmia induction with and without isoproterenol was undertaken until all inducible organized atrial arrhythmias had been mapped an ablated.

### Data collection and analysis

For each patient, we recorded age, gender, duration of AF or atrial flutter, prior antiarrhythmic drug therapy, CHADS_2_ and CHA_2_DS_2_-VAS_C_ score, cardioversions, body mass index, left atrial (LA) size, prior strokes/TIA’s, and the presence of hypertension, coronary artery disease, and dilated cardiomyopathy. A successful ablation was defined as no AF, atrial flutter, or tachycardia lasting more than 30 s off of all antiarrhythmic drugs after a 3-month blanking period.

### Follow-up

Some patients were treated with antiarrhythmic drugs and/or cardioverted during the 3 months following the ablation. Patients sent daily transtelephonic ECG strips for 1–3 months after ablation and were seen at 3 and 12 months, at which time 7–14 days of continuous monitoring using a ZIO patch (iRhythm Technologies, Inc., San Francisco, CA) was done. Thereafter, patients were seen directly or contacted by phone at least annually and arrhythmia records were obtained from the hospitals and referring physicians. ECG recorders were reissued for any arrhythmia symptoms. Pacemaker AF data were utilized when available.

### Data analysis

Continuous data were described as mean ± standard deviation and counts and percent if categorical. Kaplan-Meier curves were generated for freedom from all atrial arrhythmias and for freedom from atrial tachycardia and atrial flutter after the ablation using XLStat, 2015 (Paris, France).

## Results

### Patient population

The population consisted of 23 patients with atypical atrial flutters. The average age was 65.3 ± 8.5 years and 78% were male. The demographics are shown in Table 1. Twenty-one patients (91.3%) had undergone either prior left atrial ablation or a surgical maze procedure. The 21 patients with a prior left atrial ablation or surgical maze procedure had a total of 82 pulmonary veins. Only 24 of the 82 (29.2%) remained isolated from their previous procedure. Only three patients (9.5%) had all pulmonary veins isolated, and three patients (14.2%) had three of four pulmonary veins isolated. The two patients who had not undergone one of these procedures included one patient with a lamin AC cardiomyopathy and severe atrial fibrosis and one with a prior aortic valve replacement. Atrial fibrillation or atrial flutter had been present for 5.7 ± 4.8 years. The average left atrial size was 4.66 ± 0.64 cm. Structural heart disease was present in 34.8% of patients.

**Table 1:** Clinical characteristics

Number of patients	23
Left atrial size (cm)	4.66 ± 0.64 (3.7–5.4)
Age (years)	65.3 ± 8.5 (47–77)
Body mass index	29.8 ± 4.5 (23.5–40.8)
Gender: female	22.0%
# without prior ablation or MAZE	2 (8.7%)
Duration of AF (years)	5.7 ± 4.8 (0.5–19)
# Drugs failed	1.56 ± 1.16 (0–4)
CHADS_2_ score	2.17 ± 1.23 (0–5)
CHA_2_DS_2_-VAS_C_ score	3.04 ± 1.66 (0–7)
Hypertension	60.8%
Prior CVA/TIA	21.7%
Prior cardioversion	73.9%
Coronary artery disease	26.1%
Dilated cardiomyopathy	8.7%

Ranges in parentheses

### Mapped arrhythmias

**Fig. 1:**
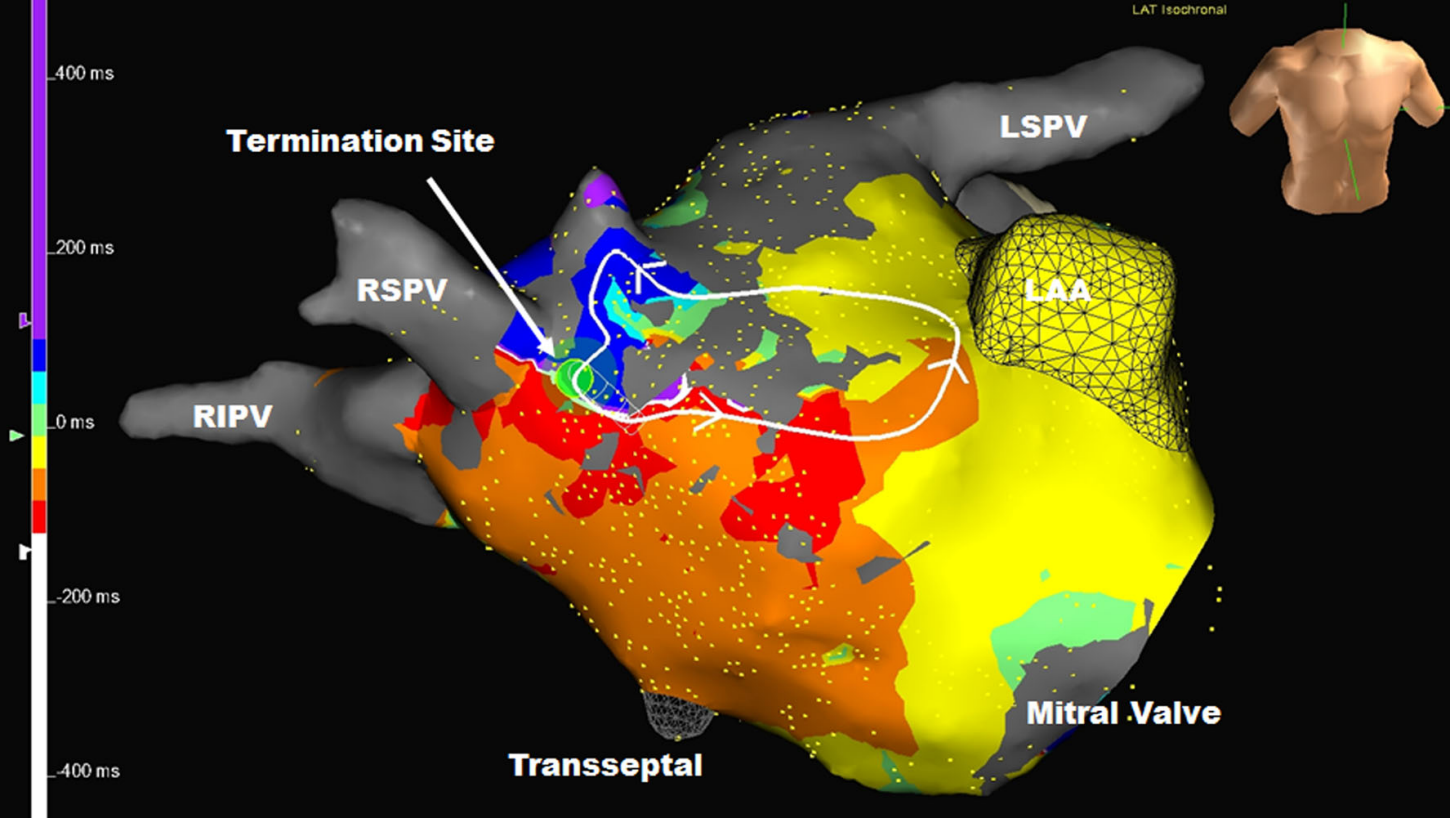
Left atrial anteroseptal flutter with an area of slow conduction between the septal scar and a previously isolated right superior pulmonary vein. (*RSPV* right superior pulmonary vein, *RIPV* right inferior pulmonary vein, *LSPV* left superior pulmonary vein, *LAA* left atrial appendage). See Appendix 1 for the propagation map of this atrial flutter

**Fig. 2:**
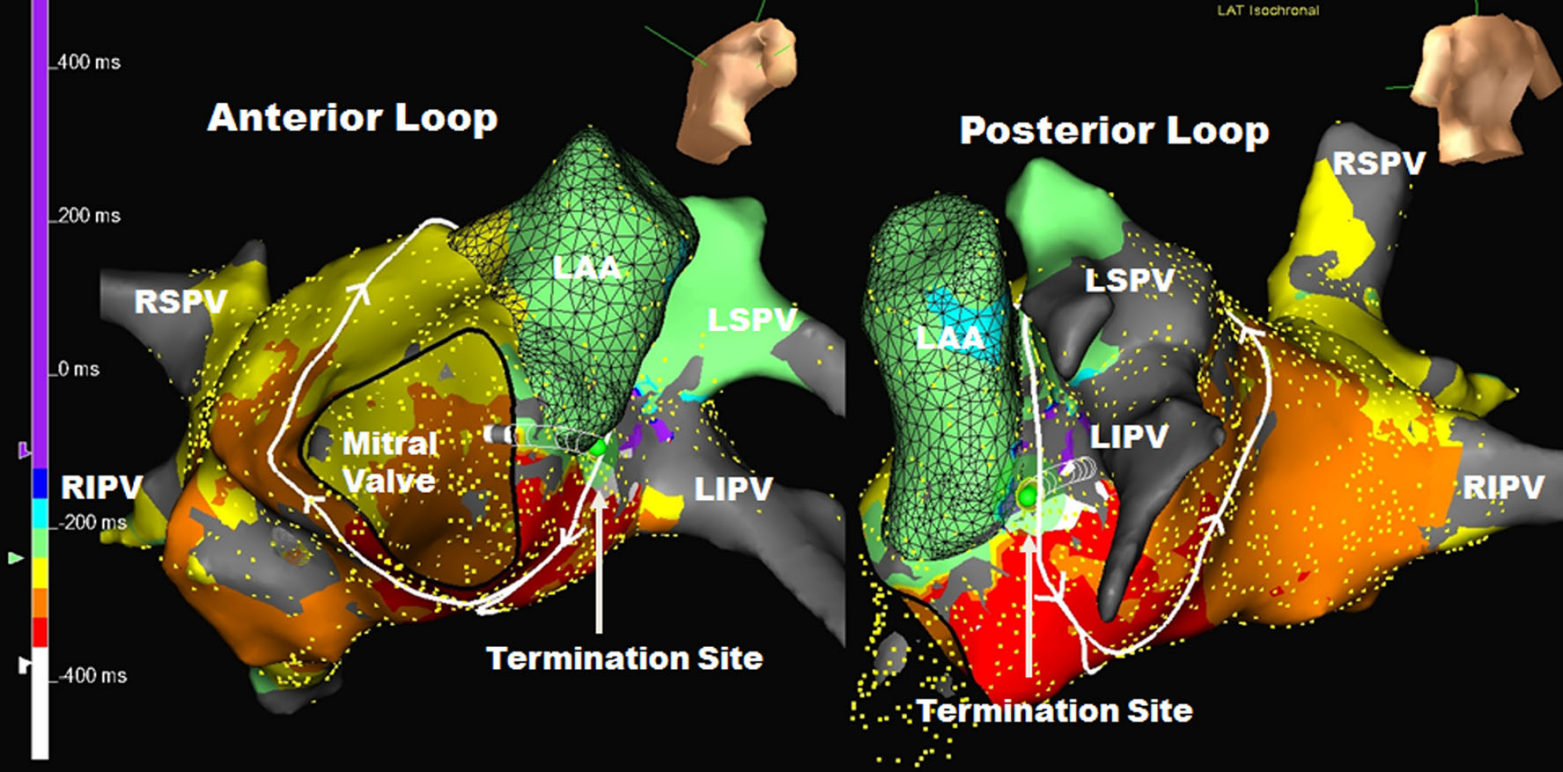
Double loop left atrial flutter with anterior loop going clockwise along the inferior mitral annulus and up the septum and the posterior loop going counterclockwise around the left pulmonary veins with a common segment of slow conduction between the left atrial appendage and the left inferior pulmonary vein. (*LIPV* left inferior pulmonary vein, other abbreviations as in Fig. 1). See Appendix 2 for the propagation map of this atrial flutter

**Fig. 3:**
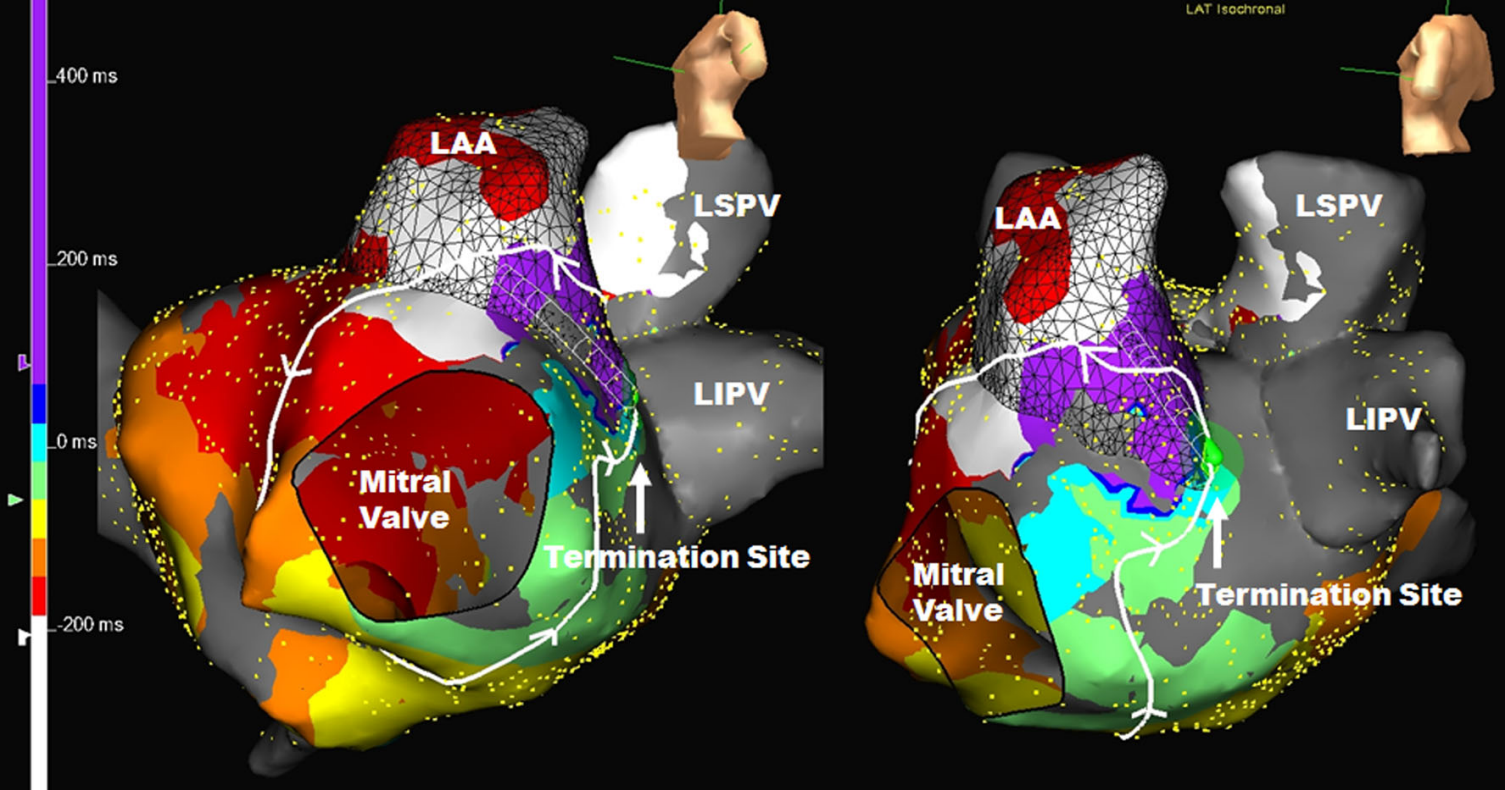
Complex mitral isthmus flutter. Much of the atrial flutter circuit is around the mitral valve. However, the circuit deviates toward the critical area of slow conduction between LAA and the LIPV. It then returns to the mitral valve via conduction through the LAA. Abbreviations as in Figs. 1 and 2. See Appendix 3 for the propagation map of this atrial flutter

A total of 31 sustained and organized atrial arrhythmias were either present clinically or induced during the study. These were macroreentrant arrhythmias in 97% and microreentrant or focal in 3%. The left atrium was involved in 30 of 31 (97%) of the arrhythmias. The average cycle length for the atrial arrhythmias was 267 ± 49 ms (range 206–444). The total length of the atrial flutter circuit for the 30 macroreentrant arrhythmias was 138 ± 38 mm (range 35–187). Each of the macroreentrant arrhythmias had a distinct area of slow conduction. The length of the area of slow conduction was 16 + 6 mm (range 6–29), and the conduction time through the slow zone was 92 ± 49 (range 33–247) ms. The length of the remainder of the atrial flutter circuit was 122 ± 36 mm (range 29–164), and the conduction time in the remaining zone was 175 ± 45 (range 88–256) ms. The location of the area of slow conduction or microreentry circuit was right atrium = 1, LA roof = 5, LA anteroseptal = 4 (Fig. 1 and Appendix 1), LA posterior = 2, LA/pulmonary vein ridge = 2 (Fig. 2 and Appendix 2), complete mitral isthmus = 4, complex mitral isthmus (where part of the circuit was around the mitral isthmus but the rest was around other parts of the atrium) = 11 (Fig. 3 and Appendix 3), and involved reentry going in and out of one of the pulmonary veins in 2.

### Mapping data and arrhythmia termination

The total number of points collected for each atrial arrhythmia mapped was 3815 ± 2576 (357–10,069). The total number of points utilized to create the map was 1273 ± 697 (range 259–2959). The difference in points collected and points used was due to the exclusion of points not in close proximity to the 3D geometry shell. For patients already in atrial flutter at the start of the case, the time from initiation of geometry creation to first RF delivery was 24 ± 11 min. For patients where the arrhythmia was induced after the geometry was created, the time from the start of the map to the first RF energy delivery was 20 ± 11 min for each flutter mapped. Twenty-eight of 31 atrial flutters terminated when ablating the slow zone or microreentry or focal site (90.3%). Nineteen of 31 (61.3%) converted directly to sinus rhythm. Seven of 31 (22.6%) changed into another atrial flutter circuit which was mapped and then terminated directly to sinus rhythm with additional ablation. Two of 31 (6.5%) degenerated from atrial flutter into atrial fibrillation during ablation of the area of slow conduction and required cardioversion. Those atrial flutters could not be reinduced. The median time from first RF to termination of the arrhythmia was 64 s (range 3–2134 s). A single patient with the extremely long time of 2134 s to termination of the arrhythmia ultimately had their arrhythmia terminated a few millimeters from where we started the first ablation. The time from first RF to atrial flutter termination was less than 1 min in 13 of 27 cases (48.1%) and was less than 4 min in 21 of 27 (78%). There were no procedural complications in any patients.

### Follow-up

**Fig. 4:**
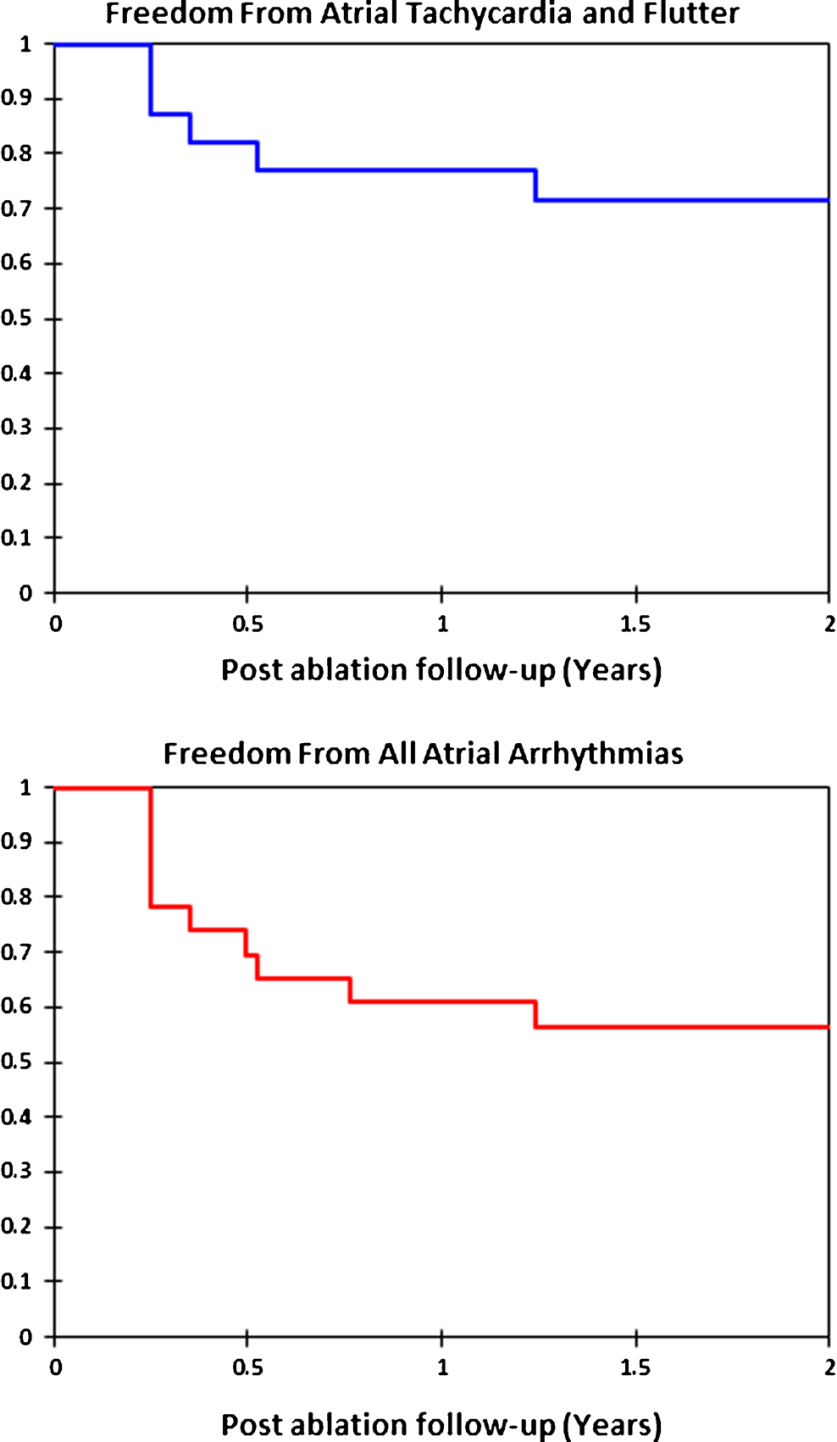
Kaplan-Meier curves for freedom from atrial tachycardia and flutter (*top panel*) and freedom from all atrial arrhythmias (*bottom panel*) following ablation for atypical atrial flutters

Figure 4 shows the Kaplan-Meier follow-up curve for freedom from atrial tachycardia and atrial flutter(top panel) and freedom from all atrial arrhythmias lasting more than 30 s (bottom panel) off of antiarrhythmic drugs following ablations for atypical atrial flutter. At 1 year, 77% were free of atrial tachycardia and atrial flutter and 61% were free of all atrial arrhythmias. Among the 10 patients who had atrial arrhythmia recurrence, the failure rhythm was atrial fibrillation in 4 patients and atrial flutter in 6 patients. One of the patients had a repeat procedure, and the atrial flutter was utilizing a different circuit than the one ablated at the index procedure. For the other five patients, as best we could determine from available ECGs, the atrial flutters did not appear to be the ones we had ablated at the index procedure. However, without detailed intracardiac mapping, it is difficult to be certain that these were indeed different atrial flutters. Two of the patients who had arrhythmia recurrence off of antiarrhythmic drugs were free of arrhythmias on an antiarrhythmic drug.

## Discussion

The main finding of this study is that it is possible, using only UHD-ASM, to map the entire arrhythmia circuit for most atypical atrial flutters and to successfully ablate them by targeting an area of slow conduction. In the past, mapping these arrhythmias has been done with older and less accurate mapping systems, limiting the number of data points which could be collected, and using larger tip ablating catheters with widely spaced bipolar electrodes resulting in poor resolution. Entrainment mapping has been utilized as an aid to determining the location of the atrial flutter circuit. However, entrainment mapping has a number of limitations for mapping these arrhythmias. The morphology of the atrial flutter on the surface ECG is often very low amplitude and it is difficult to compare the entrained surface lead morphology to the clinical morphology. In addition, measurement of the post pacing interval can be difficult and, at times, entrainment pacing attempts can either change or terminate the arrhythmia which is being mapped. It may be difficult to pace in areas of atrial scar which are part of the atrial flutter circuit or pacing may only be accomplished with higher outputs that could distort local signals. Although entrainment can give an idea of whether or not they are pacing from within the atrial flutter circuit, it provides relatively little information with regard to the optimal place to perform ablation to terminate and eliminate the arrhythmia.

In most instances, atypical atrial flutters can have an UHD-ASM collected in a very timely manner and the maps do not require any special catheters, other than a standard circular mapping catheter. When logistically possible, it would seem preferable to bring these patients for ablation when they are in their clinical atrial flutter. This eliminates the possibility of not being able to induce the clinical atrial flutter or mapping an induced non-clinical atrial flutter. If the patient is in the arrhythmia at the start of the case, these maps can be obtained at the same time one is creating the 3D geometry shell. Whatever additional time is required to create these high density maps is ultimately made up for by the relatively short time it frequently takes to effectively ablate them.

### Comparison to prior 3D mapping

There have been prior reports of 3D mapping and ablation of LA flutters. One of the earliest by Jais et al. [3] evaluated 18 atrial flutters in 17 patients. The mechanisms of the atrial flutters were similar to our findings. Entrainment mapping was also used to define the atrial flutter mechanism and circuit. That study required 39 ± 33 min of RF delivery to terminate the atrial flutters. In contrast, using newer UHD-ASM, we were able to terminate atrial flutters in a significantly shorter time. Our median total time elapsed from the start of first RF delivery to atrial flutter termination was only 64 s, likely due to a more accurate definition of the entire atrial flutter circuit and the critical areas of slow conduction for targeting RF energy application. Another study [7] of left atrial arrhythmias also showed a preponderance of macroreentry as the mechanism. That study relied heavily on entrainment mapping as well as activation sequence mapping to define the tachycardia circuit. We found that with UHD-ASM, entrainment mapping was rarely, if ever, required to identify the arrhythmia mechanism, circuit, and target area for ablation.

### Importance of closely spaced mapping electrodes

A recent report by Anter et al. [13] emphasized the importance of using smaller and closely spaced electrodes to create these high-density atrial activation maps. They compared maps created with a standard irrigated tip ablation catheter with 3.5 mm distal electrodes separated by 2 mm from a proximal 2 mm electrode with a center-to-center inter-electrode spacing of 4.75 mm to those obtained with 1 mm electrodes and 2 mm inter-electrode spacing with a 3 mm center-to-center inter-electrodes pacing. When mapping healthy atrial tissue, there was little difference between the two mapping catheters. However, in atrial scar, the larger electrodes with greater spacing resulted in an overestimation of the area of scar and only allowed for identification of 68 + 32% of the tachycardia cycle length compared to identification of 92 + 8% of the tachycardia cycle length with the smaller electrode spacing. Our mapping electrode had 1 mm electrodes separated by 1 mm for a center-to-center internal electrode spacing of 2 mm. This should provide even better resolution for mapping of the tachycardia than the electrodes used by Anter et al. [13].

### Long-term outcomes

Although we were highly effective in mapping and ablating the clinical as well as all induced arrhythmias, the long-term outcome in these patients with regard to freedom from all arrhythmias was relatively disappointing. While our definition of procedural failure includes the occurrence of atrial fibrillation, others [5] have only included the recurrence of atrial tachycardias after this type of ablation. Using this definition, our 1-year success rate would have been 77% rather than 61%. As best we could determine from available ECG tracings, none of the mapped and ablated arrhythmias came back. However, these patients had highly diseased atria with significant intrinsic scar and scar created at previous procedures which probably explains the return of other atrial arrhythmias during follow-up.

### Limitations

This was a single-center study. Patients did not have implantable loop recorders after ablation, so we undoubtedly missed some episodes of asymptomatic atrial arrhythmias during follow-up.

## Conclusions

Using rapidly acquired UHD-ASM, the entire AAF circuit as well as a target ASC can be identified. Most AAF were LA macroreentry. No entrainment mapping was required. Ablation of the ASC or microreentry focus directly terminated or eliminated AAF in 90.3% of arrhythmias.

## Electronic supplementary material

Below is the link to the electronic supplementary material.

ESM 1 (MPG 3101 kb)

ESM 2 (MPG 3428 kb)

ESM 3 (MPG 6052 kb)

## References

[1] Rytand DA (1966). The circus movement (entrapped circuit wave) hypothesis and atrial flutter. Ann Int Med.

[2] Stibitz GR, Rytand DA (1968). On the path of the excitation wave in atrial flutter. Circulation.

[3] Jais P, Shah DC, Haissaguerre M, Hocini M, Peng JT, Takahashi A (2000). Mapping and ablation of left atrial flutter. Circulation.

[4] Jais P, Matsuo S, Knecht S, Weerasooriya R, Hocini M, Sacher F (2009). A deductive mapping strategy for atrial tachycardia following atrial fibrillation ablation: importance of localized reentry. J Cardivasc Electrophysiol.

[5] Coffey JO, d’Avila A, Dukkipati S, Danik SB, Gangireddy SR, Koruth JS (2013). Catheter ablation of scar-related atypical atrial flutter. Europace.

[6] Chae S, Oral H, Good E, Dey S, Wimmer A, Crawford T (2007). Atrial tachycardia after circumferential pulmonary vein ablation of atrial fibrillation. J Am Coll Cardiol.

[7] Patel AM, d’Avila A, Neuzil P, Kim SJ, Mela T, Singh JP (2008). Atrial tachycardia after ablation of persistent atrial fibrillation. Circ Arrhythmia Electrophysiol.

[8] Santucci PA, Varma N, Cytron J, Akar JG, Wilbur DJ, Al Chekakie MO (2009). Electroanatomic mapping of postpacing intervals clarifies the complete active circuit and variants in atrial flutter. Heart Rhythm.

[9] Chugh A, Oral H, Good E, Han J, Tamirisa K, Lemola K (2005). Catheter ablation of atypical atrial flutter and atrial tachycardia within the coronary sinus after left atrial ablation for atrial fibrillation. J Am Coll Cardiol.

[10] Waldo AL (1997). Atrial flutter: entrainment characteristics. J Cardiovasc Electrophysiol.

[11] Winkle RA, Mead RH, Engel G, Patrawala RA (2011). Long term results of atrial fibrillation ablation: the importance of all initial ablation failures undergoing a repeat ablation. Am Heart J.

[12] Winkle RA, Mead RH, Engel G, Kong MH, Patrawala RA (2014). Peri-procedural interrupted oral anticoagulation for atrial fibrillation ablation: comparison of aspirin, warfarin, dabigatran, and rivaroxaban. Europace.

[13] Anter E, Tschabrunn CM, Josephoson ME (2015). High-resolution mapping of scar-related atrial arrhythmias using smaller electrodes with closer interelectrode spacing. Circ Arrhyth Electrophysiol.

